# Experimental and Numerical Research of 3D DLP-Printed Solid and Voronoi PLA Resin Specimens Under Tensile and Bending Loads

**DOI:** 10.3390/polym17091180

**Published:** 2025-04-26

**Authors:** Zorana Golubović, Jovan Tanasković, Aleksa Milovanović, Božica Bojović

**Affiliations:** 1 Faculty of Mechanical Engineering, University of Belgrade, 11120 Belgrade, Serbia; jtanaskovic@mas.bg.ac.rs (J.T.); bbojovic@mas.bg.ac.rs (B.B.); 2 Innovation Center of Faculty of Mechanical Engineering, 11120 Belgrade, Serbia; amilovanovic@mas.bg.ac.rs

**Keywords:** additive manufacturing, 3D printing, PLA-like resin, solid, Voronoi, digital light processing (DLP), tensile testing, three-point bending, numerical simulation, microscopy

## Abstract

Additive manufacturing (AM), especially vat photopolymerization processes such as digital light processing (DLP), enables the production of highly detailed and complex geometries with precise material structure control. In this study, the influence of internal structure on the mechanical properties of PLA resin specimens produced using a DLP 3D printer is investigated. Two designs were analyzed: a fully solid structure and a shell with a Voronoi pattern. Tensile and bending tests revealed that solid specimens exhibited higher strength, while Voronoi structures performed better under bending loading despite lower load-bearing capacity due to their porosity ratio. The developed numerical model, analyzed through different numerical simulations using the Ansys 2025R01 Software package and validated by experimental results, showed a strong correlation between experimental and numerical results that confirmed the reliability of the developed models for preliminary design verification. These models hold significant potential for the design of mechanical and biomedical components, including orthopedic immobilization devices. Microscopic analysis revealed brittle fracture in solid specimens with striations and bubble-shaped irregularities, while Voronoi specimens exhibited fragmented surfaces with clean, brittle failure along structural voids. Based on the results obtained, this research demonstrates how additive manufacturing enables the optimization of mechanical properties and material efficiency through precise control of internal structures. In the future, validated numerical models can be used to check the preliminary designs of different components, which will significantly reduce development costs.

## 1. Introduction

In an age of rapid technological progress, one of the most transformative innovations is the advance of additive manufacturing (AM), i.e., 3D printing. This technology has revolutionized traditional production methods by enabling the layer-by-layer production of complex geometries with minimal material waste [1]. Unlike traditional subtractive processes, where raw materials are cut and shaped, additive manufacturing creates objects directly from digital models, allowing for unprecedented design flexibility and customization [2]. The importance of this field extends to various industries, including aerospace, automotive manufacturing, and healthcare, where the production of lightweight structures, patient-specific implants, and intricate components that would be difficult or impossible to realize using conventional methods is possible [3]. Continuous advances in materials, precision, and efficiency are expanding the role of 3D printing in modern engineering and manufacturing [4,5].

Among AM techniques, vat photopolymerization encompasses several additive manufacturing techniques, including stereolithography (SLA), digital light processing (DLP), masked stereolithography (MSLA), two-photon lithography (2PL), and continuous liquid interface production (CLIP) [6,7]. These processes all use UV light to cure liquid resin and enable the precise layer-by-layer production of 3D objects. The DLP process has attracted attention due to its ability to improve both printing speed and accuracy and thus represents a valuable advance in 3D printing technology. Unlike the SLA process, where the resin is cured in spots, the DLP process cures entire layers at once, which significantly increases efficiency. This is achieved by using digital micromirror devices [8] or dynamic liquid crystal masks [9]. With a typical resolution between 1 and 10 µm, DLP printers can achieve speeds of up to 103 mm per hour [10]. Studies suggest that DLP-printed parts may have better mechanical properties compared to SLA, especially in terms of flexural strength and stiffness, although material formulations and processing conditions play an important role [11]. To ensure the reliability and applicability of vat photopolymerized printed parts, it is essential to evaluate their mechanical properties, as these have a direct impact on their structural integrity and functional performance. Understanding how factors such as material composition, curing conditions, and geometric design affect strength, stiffness, and durability can help optimize them for engineering and biomedical applications.

This research aims to give a comprehensive understanding of how different geometric designs, specifically solid vs. Voronoi lattice structures, affect the mechanical performance of DLP-printed PLA resin parts.

Solid structures in DLP printing generally offer high strength and durability, as there are no internal voids, resulting in uniform stress distribution under mechanical loading [12]. Although solid structures offer high strength due to the even distribution of stress, they are only suitable for lightweight construction applications to a limited extent due to their material consumption and weight [13]. In contrast, Voronoi lattice structures offer an efficient design alternative that optimizes material consumption while maintaining mechanical integrity, as shown by studies on their parametric design and mechanical behavior [14]. The Voronoi design, inspired by natural cellular structures, enables a high strength-to-weight ratio and energy absorption properties, making them suitable for impact-resistant applications [15,16]. Studies have shown that Voronoi lattices can significantly improve mechanical performance, especially under dynamic loading conditions, while reducing overall material waste [17]. Considering the advantages of Voronoi lattice designs, research was necessary for the implementation of PLA resins to evaluate their mechanical performance and structural efficiency.

Polylactic acid (PLA), a biodegradable polymer made from renewable sources like cornstarch and sugarcane, is widely used in 3D printing due to its ease of processing, environmental benefits, and favorable mechanical properties [18,19]. While thermoplastic PLA filaments are commonly employed in fused deposition modeling (FDM), liquid PLA-based resins are gaining attention in digital light processing (DLP) and stereolithography (SLA) due to their high-resolution capabilities [20]. These resins contain photoinitiators and crosslinking agents that enable them to solidify under UV irradiation [21,22]. PLA resins are known for their ability to produce high-resolution prints with smooth surfaces, making them ideal for applications that require high precision. However, their tendency to degrade and lose mechanical strength over time highlights the need for strategies to improve their durability and ensure they remain reliable in real-world use [23]. In contrast to thermoplastic PLA, which retains a certain ductility, photopolymerized PLA resins have a highly cross-linked structure, resulting in increased stiffness and strength but lower elongation at break. This inherent brittleness remains a limitation, although recent studies have explored ways to improve toughness by blending PLA resins with flexible oligomers or reinforcing them with nanomaterials such as cellulose and graphene [15]. The study by Efstathiadis et al. has shown that biomimetic Voronoi lattices inspired by the sea urchin stereome and made from PLA composites show a promising balance between light weight and mechanical strength, making them suitable for applications requiring optimized structural efficiency [14]. The other study concludes that printed Voronoi structures made from PLA, inspired by the shell microstructure of *Paracentrotus lividus*, exhibit improved mechanical properties and energy absorption capabilities, making them suitable for lightweight and protective applications in engineering and design [24].

When evaluating the mechanical properties of 3D-printed polymers, tensile and three-point bending tests are often performed, as they provide different but complementary information about the behavior, structural integrity, and properties of the material. The tensile test measures the material’s response to uniaxial stress and provides important information about properties such as tensile strength, modulus of elasticity, and elongation at break. These parameters are important to understand how the polymer will behave under tension in real-world applications. Tensile testing is particularly important to evaluate the anisotropy of 3D-printed materials, as mechanical properties can vary depending on print orientation and layer bonding [25,26]. On the other hand, three-point bending tests evaluate the flexural strength and flexural modulus of materials, reflecting their ability to resist deformation under bending load. This test is crucial for applications where the polymer specimen may be subjected to bending or flexural stresses, such as beams, frames, or panels. In addition, three-point bending tests can also reveal the effects of printing defects, such as voids or incomplete interlayer bonding, which are critical for determining the durability of 3D-printed components [27,28]. The combination of tensile and three-point bending tests enables a comprehensive mechanical characterization of 3D-printed polymers. Tensile tests focus on material properties in tension, while three-point bending evaluates structural performance in flexure. Together, they provide an integrated understanding of mechanical behavior and enable optimization of printing parameters, material selection, and design for improved performance and reliability in specific applications [29,30].

The development of numerical models is essential for carrying out various analyses prior to the production and final testing of prototypes, significantly reducing research costs. By accurately defining material models and analysis settings that are validated based on experimental results, researchers can efficiently evaluate preliminary designs under various loading conditions within a short time frame, eliminating the need for extensive experimental testing. In addition, numerical analyses can be performed on scale models during the development phase to obtain more meaningful parameters for the final design. It should be noted that the final full-scale prototype test is necessary as it represents the final approval for the design of each new element, sub-assembly, and assembly.

By combining numerical modeling and microscopic analysis, researchers gain a more comprehensive understanding of material behavior, allowing them to improve their designs based on both calculated predictions and real-world observations. In order to analyze defects, evaluate failures, and assess both the material composition and the mechanical properties of a component, researchers carefully examine the cross-sectional images captured by microscopy. These images provide important insights into the internal structures and reveal inconsistencies, cracks, or weak points that can affect performance and durability. By examining these micrographs, engineers and scientists can identify the causes of defects, optimize manufacturing processes, and improve the overall quality and reliability of materials used in various applications [31].

In our research, mechanical tests are carried out in a controlled laboratory environment with standardized specimens. These specimens are designed to replicate the material properties and structural characteristics relevant to biomedical applications. By using precise experimental methods, we aim to evaluate the mechanical behavior of these materials under controlled conditions to ensure reproducibility and accuracy. The findings from these lab-scale experiments will provide versatile data for applications where both strength and material efficiency are key. It will also provide a basis for future research on full-scale biomedical immobilization devices, such as splints, braces, and orthopedic supports intended for use in rehabilitation after fractures and injuries. This approach enables systematic evaluation of material performance prior to clinical use.

This research combines experimental mechanics, numerical modeling, and microstructural analysis to evaluate the mechanical efficiency of Voronoi lattice geometries in DLP-printed PLA resin parts. While previous research studies have investigated lattice structures or material formulations separately, this work integrates them into a biomedical context to optimize both strength and material utilization. The approach provides important data for the design of next-generation immobilization devices that are lightweight, customizable, and mechanically reliable.

## 2. Experimental Research

### 2.1. Specimen Preparation

A commercially available PLA-like resin (eSUN, Shenzhen, China) was selected for this research. The geometry of the solid specimens was designed using the 32. SolidWorks Premium 2024 SP05 software package [32] in accordance with the ISO 527-2 [33] standard for tensile tests and the ISO 178:2019 [34] standard for three-point bending tests. The Voronoi designs were created using Grasshopper for Rhino 5.0 to ensure consistency of geometry. The finished model was exported as an STL file and prepared for printing using ChiTuBox V1. 9.4 slicing software (Shenzhen, China). A total of ten specimens were produced, five for tensile tests and five for three-point bending tests. The solid specimens were printed with a 90° orientation, a grid infill pattern, and an infill density of 100, and other printing parameters are given in Table 1. They were produced using the Creality LD-002R DLP printer (Creality, Shenzhen, China) under identical printing conditions, with the only variation being the internal structure.

Specimens underwent cleaning with alcohol followed by exposure to LED 405 nm wavelength light for additional UV curing. Curing was performed for 4 min in a closed chamber with a rotational stage. No polishing was performed to remove any remaining support material.

### 2.2. Mechanical Testing

**Figure 1:**
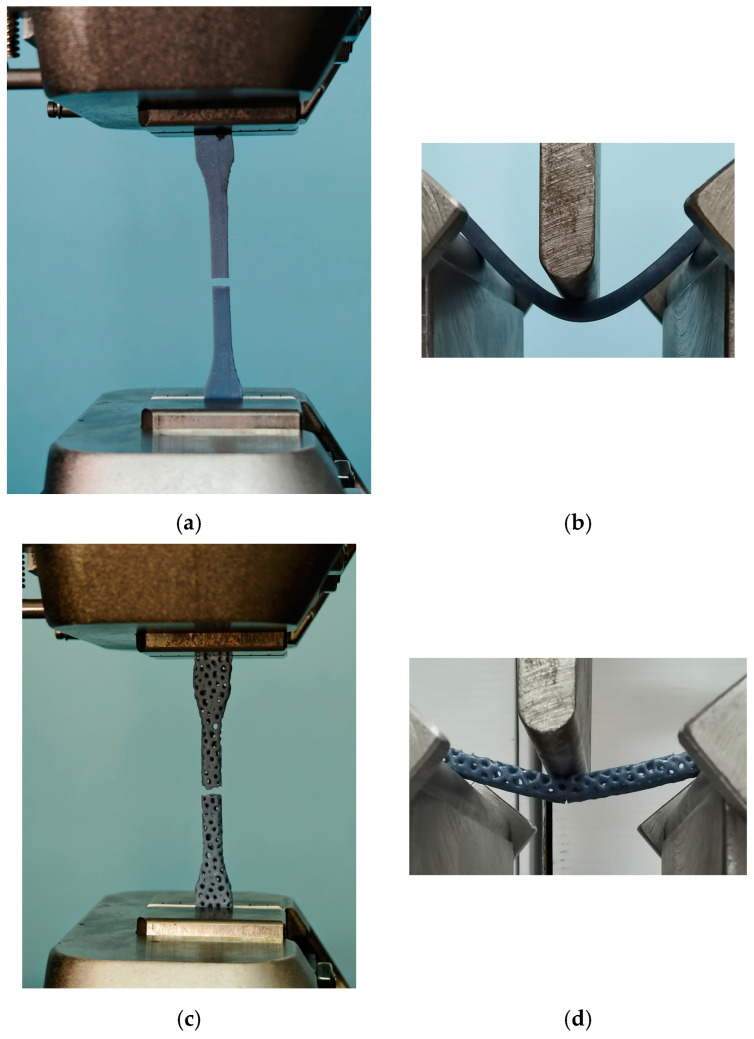
Mechanical testing of specimens: (**a**) tensile solid, (**b**) bending solid, (**c**) tensile Voronoi, and (**d**) bending Voronoi.

Mechanical testing was performed using a Shimadzu AGS-X universal testing machine (Shimadzu Corp., Kyoto, Japan), which can realize a maximum load of 100 kN (Figure 1). The test was conducted at a speed of 1 mm/min, following the relevant standards [33,34]. For each mechanical test, five specimens were analyzed, and the corresponding average stress vs. strain curves were generated. Data processing and analysis were carried out, incorporating results from both mechanical testing procedures associated with the 3D printing processes.

### 2.3. Microscopy

To assess the internal structure of the material at fracture places after mechanical testing, optical microscopy was employed as a supplementary method. Micrographs were captured using the Mustool G600 Digital Portable 2D Microscope (Shenzhen, China). The magnification varied between 50× and 100×, depending on the characteristics of the specimen and the nature of the crack. This technique provided valuable insight into microstructural surface morphology, material cracking, the influence of trapped air, and surface finish [35]. By analyzing these parameters deeper understanding of structural changes occurring during the printing process is obtained, together with their impact on mechanical performance and potential strategies for optimizing both fabrication and post-processing methods.

## 3. Numerical Investigations

**Figure 2:**
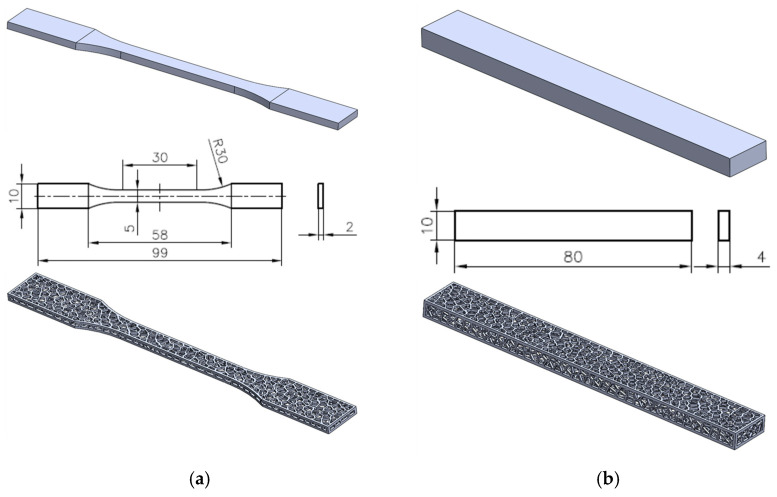
Three-dimensional model of solid and Voronoi specimens: (**a**) tensile and (**b**) three-point bending.

In this paper, the finite element model (FEM) is used to simulate the tensile and bending testing of 3D-printed PLA solid and Voronoi specimens. The experimental processes are modeled as quasi-static, using solid geometries. The 3D solid specimens were prepared using the SolidWorks software package according to the required dimensions from standards, as shown in Figure 2 [33,34]. In the experimental tests presented previously (the experimental part of this work), specimens of identical shape and dimensions were used for both the solid and Voronoi configurations.

The main objective is to validate the developed FEM for the development of PLA resin components/parts and assemblies. In this way, the number of full-scale tests required for final verification is reduced, significantly reducing development costs and offering the possibility of analyzing more details or parameters useful for the design of different types of PLA parts in the future, especially using the Voronoi pattern in 3D printing. The key to the evaluation is the stress vs. strain curve and the deformation shape before and after the tests. The nonlinear FE analysis was performed due to the large deformations and frictional contact behavior between the specimen, the steel supports, and the loading edge during testing.

### 3.1. Finite Element Model (FEM)

**Figure 3:**
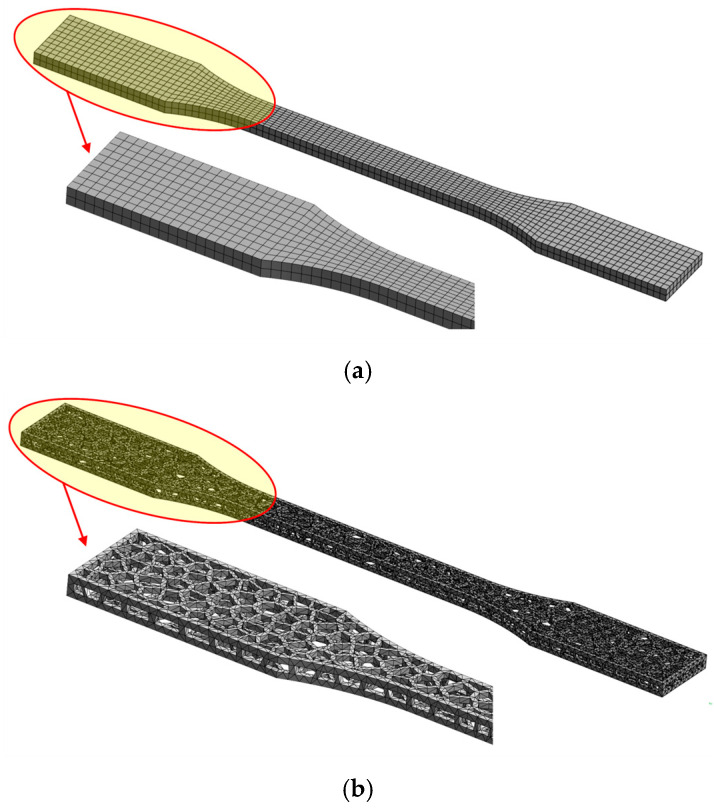
Numerical model meshed: (**a**) tensile solid specimen and (**b**) tensile Voronoi specimen.

**Figure 4:**
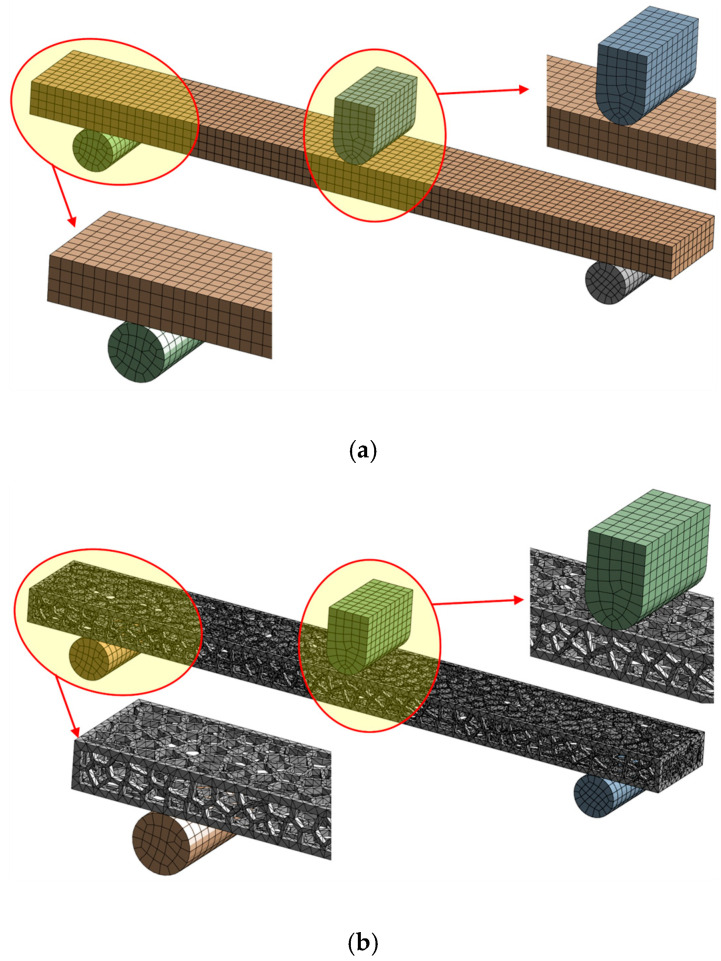
Numerical model meshed: (**a**) bending solid specimen and (**b**) bending Voronoi specimen.

The finite element models were created from a 3D CAD model using the ANSYS 2025R01 software package [36]. Tensile and bending 3D models were divided into hexahedral and tetrahedral elements, forming a finite element mesh with a maximum element size of 1 mm (Figure 3 and Figure 4). Tetrahedral elements were used for Voronoi specimens due to their complex stochastic geometries.

To minimize node penetration and oscillation of contact forces, elements of the same size were applied to cover the contact surfaces between steel supports and both solid and Voronoi specimens. Previous experience indicates that using smaller elements increases computational time without significantly improving the accuracy of stress and deformation calculations.

### 3.2. Contact Algorithm and Boundary Conditions

**Figure 5:**
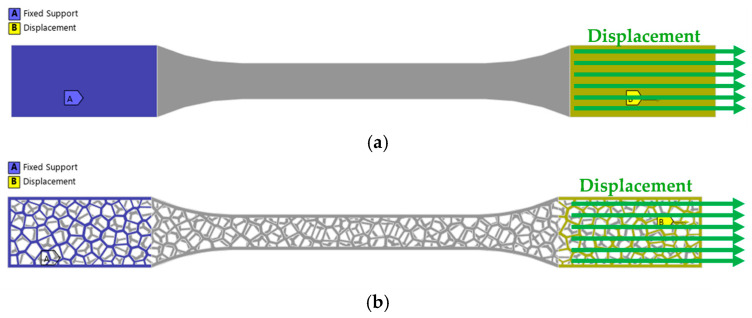
Boundary conditions—tensile: (**a**) solid specimen and (**b**) Voronoi specimen.

A simulation of the tensile and bending testing was conducted in ANSYS [36] using a frictional contact type with a friction coefficient of 0.2–0.4 [37,38,39]. A value of 0.2 is used between steel supports (stainless steel-polished surfaces) and the specimen, while a value of 0.4 is used between the loading edge (structural steel) and the specimen. The program-controlled frictional contact algorithm included automatic pinball region detection and adjust-to-touch interface handling. Numerical simulations of tensile tests of solid and Voronoi specimens were performed with fixed constraints applied (zero-stroke boundary conditions) at the surfaces of contact of the specimens and the fixed jaws of the testing machine. Stroke was applied on the other side of the specimens, i.e., at the surfaces of contact of the specimens and the moving jaws of the testing machine (Figure 5).

**Figure 6:**
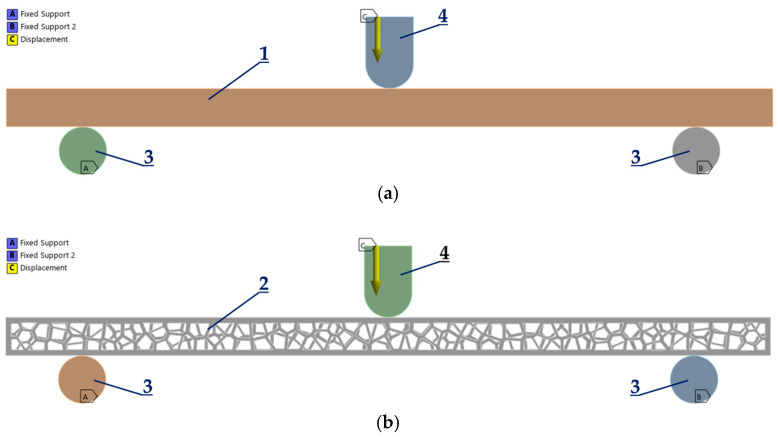
Boundary conditions—bending: (**a**) solid specimen and (**b**) Voronoi specimen.

The finite element analysis (FEA) was carried out on bending tests on both solid and Voronoi specimens (Figure 6, items 1 and 2). Fixed constraints were applied at the steel supports, implementing zero-stroke boundary conditions (Figure 6, item 3), while stroke was applied at the loading edge (Figure 6, point 4), which was connected to the moving head of the testing machine, as shown in Figure 6.

The FEA applied a stroke of 5.9 mm for the tensile tests on the solid and Voronoi specimens and 16 mm for bending tests on both specimens. These values are in agreement with the experimental results.

### 3.3. Material Model of PLA Specimens

**Figure 7:**
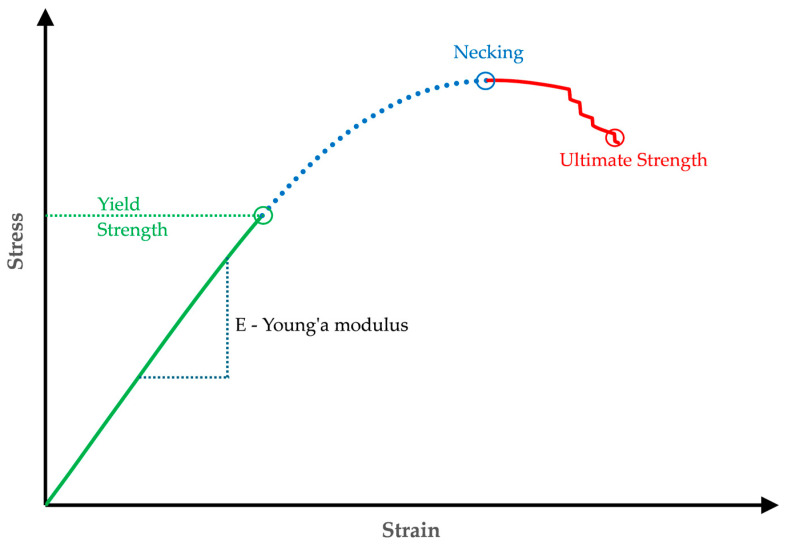
Stress vs. strain curve: multilinear hardening plasticity material model.

Due to the nonlinear analysis, which includes large deformations and the behavior of the material in tension and bending, a multilinear isotropic strain hardening plasticity model was chosen to model the PLA specimens. Figure 7 shows a typical stress vs. strain curve for PLA under uniaxial tension. This curve characterizes linear elastic deformations, where the stress is directly proportional to the strain and the slope represents the Young’s Modulus of the material. If the stress exceeds the elastic limit, known as the yield point, the response becomes nonlinear due to strain hardening. The yield strength has increased due to the hardening. The material continues to harden nonlinearly until necking occurs. Beyond this point, the stiffness decreases until the material fails at the ultimate strength (start of material fracture). The multilinear hardening model uses a piecewise linear function for the nonlinear hardening response up to necking (Figure 7, blue line) and does not support the negative slope part of the (Figure 7, red line).

To define this behavior, a distinction must be made between elastic and plastic behaviors. The former is the linear–elastic behavior defined by Young’s modulus of elasticity and Poisson’s ratio. Plastic behavior, defined by the plastic strain compared to the true stress, shows multilinear hardening. The first data point indicates the onset of plastic deformation, where the strain is “zero” and the stress is equal to the “yield strength”.

**Figure 8:**
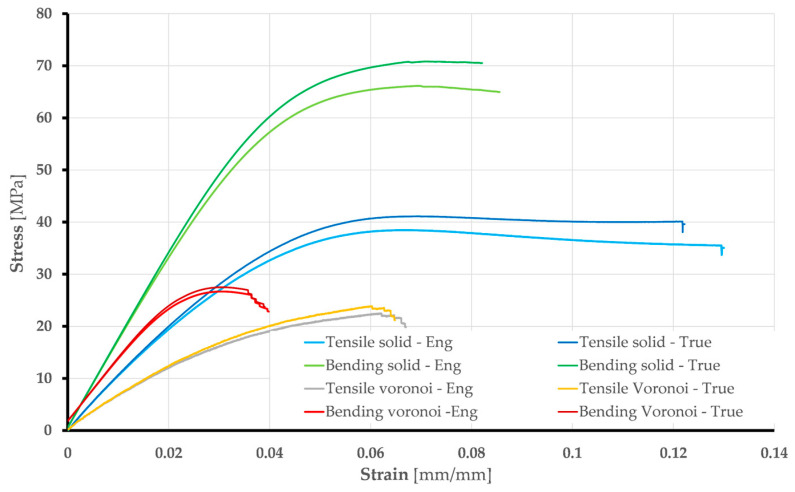
Stress vs. strain average curves: engineering and true.

These material properties were derived from experimental results, including five tensile and bending tests on solid and Voronoi specimens. The average stress vs. strain curves for each FEA type are shown in Figure 8.

Before differentiating between elastic and plastic behavior, the engineering stress vs. strain data were converted into true stress vs. strain data using the following equations:

(1) $$\sigma_{true}=\sigma_{emg}(1+\varepsilon_{eng})$$

(2) $$\epsilon_{true}=\ln\left(1+\varepsilon_{eng}\right)$$

Based on the previous explanations and taking into account the standard requirements [33,34], the values of Young’s modulus of elasticity were calculated for solid and Voronoi specimens: Tensile tests gave values of 720 MPa (Voronoi) and 1158 MPa (solid); bending tests gave values of 1212 MPa (Voronoi) and 1708 MPa (solid). Poisson’s ratio is 0.36 in both the elastic and plastic ranges. The initial yield strength is determined from the test data, which correspond to the true curve (Figure 8). The material model for the steel supports and the loading edge, which are both made of structural steel, is taken from the ANSYS Engineering database: Young’s modulus of elasticity has a value of 200 GPa, yield strength is 250 MPa, and Poisson’s ratio is 0.3. These elements were not analyzed in detail but served as a supporting tool for the simulation process.

## 4. Results and Discussions

### 4.1. Experimental Results

The Chitubox Basic V2 software is specialized 3D slicer software and has the ability to calculate the mass for 3D-printed parts according to the resin density. The mass for solid parts is called *m_solid_*, and the mass for Voronoi parts is called *m_voro_*. The mass ratio, *m_ratio_*, which represents the porosity of the Voronoi specimens compared to the solid specimens, is calculated using Equation (3). The results for the mass ratio are shown in Table 2.

*m_ratio_* = *m_voro_/m_solid_* (3)

**Figure 9:**
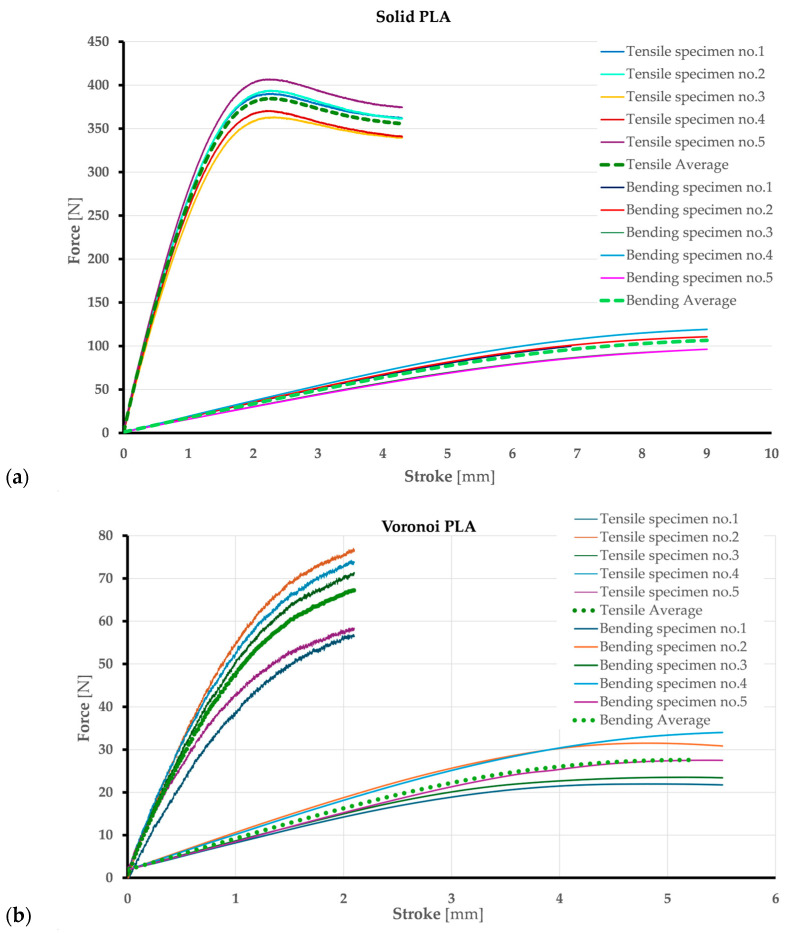
Force vs. stroke curves for tensile and three-point bending tests: (**a**) solid and (**b**) Voronoi specimens.

In this research, the mechanical properties of tension and three-point bending are determined after testing two groups of standard specimens for each type of mechanical test. The tests were performed on a group of five specimens. During the test, data were recorded using Trapezium X V1.5.0 software, showing the change in force vs. stroke. The data were recorded for five specimens per group and are shown in Figure 9. Figure 9 shows force vs. stroke diagrams including five specimens for each type of test and average curves calculated based on them.

Figure 9 shows a clear difference between the maximum force value that the solid specimens can withstand and the maximum force value for a shell with a Voronoi structure, both in the tensile test (Figure 9a) and in the three-point bending test (Figure 9b). The maximum values of force vs. stroke are shown in Table 1 for tensile and bending tests. *F_ratio_* and *stroke_ratio_* represent the force vs. stroke of the Voronoi specimen compared to the full specimen and are calculated using Equations (4) and (5) and shown in Table 2. The tensile force at the yield point for the Voronoi specimen is 0.17% of the force of the solid specimen. The tensile stroke at the point of failure for the Voronoi specimen is half the stroke for the solid specimen. The maximum three-point bending force at the yield point for the Voronoi specimen is a quarter of the maximum force recorded for the solid specimen. The bending stroke at the fracture point for the Voronoi specimen is 60% of the stroke recorded for the solid specimen.

*F_ratio_* = *F_voro_/F_solid_*, (4)

*stroke_ratio_* = *stroke_voro_/stroke_solid_* (5)

In order to compare the mechanical properties, stress vs. strain curves should be determined and used for both groups of specimens. According to the standard test for solid specimens, the raw data are converted into stresses that take into account the cross-section on which the force acts. In the case of the solid specimen, the cross-section is rectangular, while the cross-section of the Voronoi-structured specimen changes in a random pattern of struts that absorb the load.

It is obvious that Voronoi-structured specimens cannot withstand the same tensile and bending force as solid specimens, and also the fracture point is at a lower stroke value for Voronoi than for solid specimens. In terms of porosity ratio, the Voronoi pattern provides better mechanical behavior for bending loads than for tensile loads.

### 4.2. Micrographs

Microscopy was performed on PLA resin specimens after tensile and three-point bending testing to gain insight into the morphology of the fractured surfaces.

**Figure 10:**
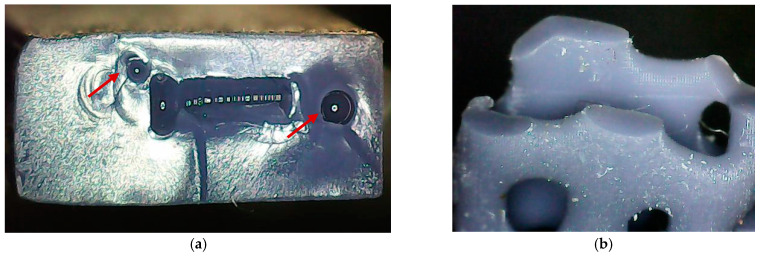
Microscopy of tested tensile fractured surfaces: (**a**) solid specimen and (**b**) Voronoi specimen.

The tensile test leads to fractures in the narrow area of all specimens. The differences between the fracture surfaces of solid and Voronoi specimens are shown in Figure 10. In Figure 10a, a clean, brittle, and straight fracture can be seen in the solid specimens, and the fracture surface has a rectangular shape. Irregularities in the shape of bubbles can be observed in the fracture surface (red arrows point to them), and the occurrence of bubbles has already been recognized as air inclusions in the shaken resin in the DLP-LCD printing process [40]. The fractured surface of the Voronoi specimen has an irregular shape and consists of several fragmented areas, which can be seen in Figure 10b. These surfaces are located along the fracture line connecting the holes of the Voronoi structures. Each of these fragmented surfaces has a clean and brittle fracture.

**Figure 11:**
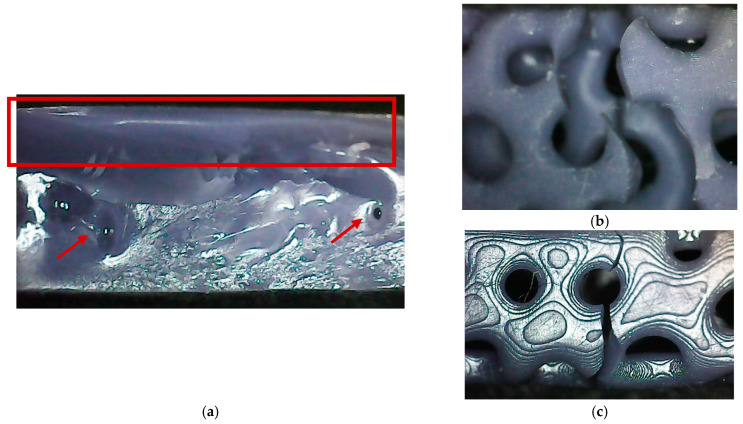
Microscopy of tested bending fractured surfaces: (**a**) solid specimen, (**b**) front view of Voronoi specimen, and (**c**) side view of Voronoi specimen.

The three-point bending did not lead to fracture in all specimens. Some of the solid PLA resin specimens remained unbroken, and all Voronoi specimens were partially broken. According to the results of FTIR spectrometry presented in [23], the PLA-like material is a UV-curing polyurethane acrylate resin. Therefore, specimens made of PLA-like resin show rubber-like behavior and high flexibility thanks to the polyurethane component. The fracture surfaces of the solid and Voronoi specimens are shown in Figure 11, and clear differences between these fractures can be seen. In Figure 11a, a brittle fracture with grooves can be seen in the main part of the cross-section of the solid specimen. After the triple bending test, the fractured specimen was divided into two interlocking parts, one of which has a step-like expansion (see red rectangle in Figure 11a), while the other part is missing the matching piece. In the fracture surface, bubble-like irregularities (indicated by red arrows) appear as trapped air. The Voronoi specimens were fractured on several surfaces (Figure 11b,c), which show a clean and brittle fracture. These fracture surfaces connect the holes of the Voronoi structures and form a curved fracture line that coincides with the position of the loading edge during the triple bending test.

The fracture morphology appears to be similar for the same type of specimen under tensile and bending loading. The solid specimen had a brittle fracture with striations and bubble-shaped irregularities. Voronoi specimens had fragmented surfaces that exhibit a clean and brittle fracture. These surfaces are located along the broken line connecting the holes of the Voronoi structures.

### 4.3. Numerical Results

**Figure 12:**
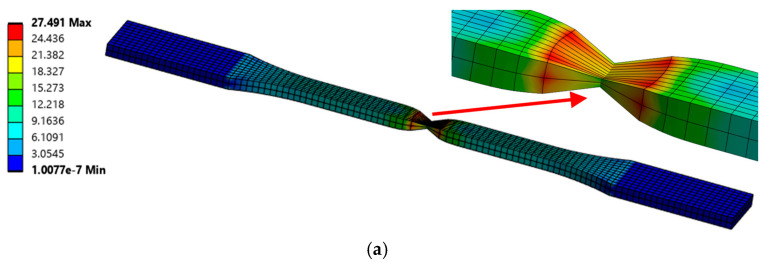

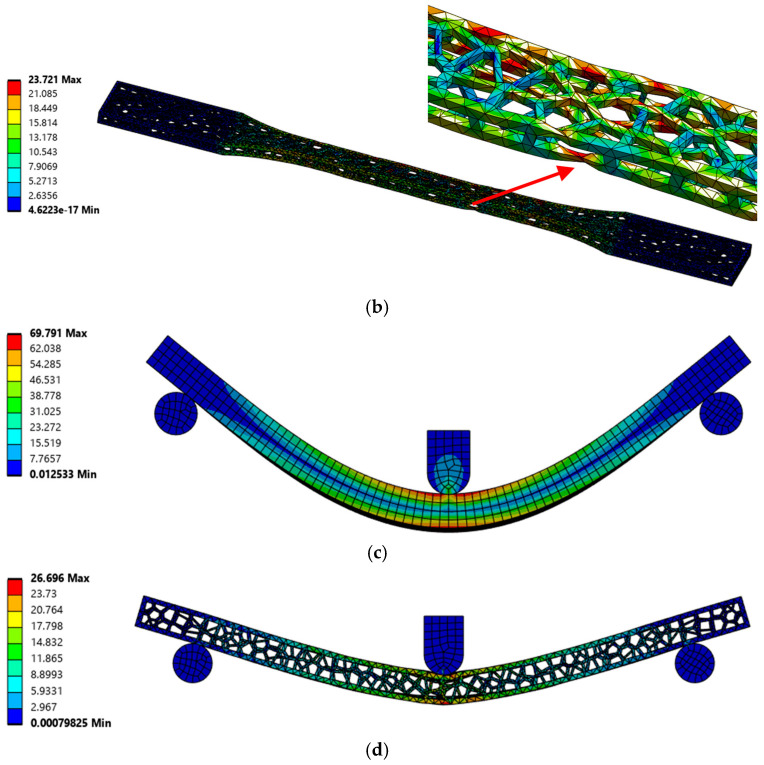
Von Mises stresses [MPa]: (**a**) tensile—solid specimen, (**b**) tensile—Voronoi specimen, (**c**) bending—solid specimen, and (**d**) bending—Voronoi specimen.

Numerical analyses were performed on two tensile and two bending specimens. The deformed specimens and the corresponding von Mises stresses, together with the onset of necking in both the solid and Voronoi specimens, are shown in Figure 12.

**Figure 13:**
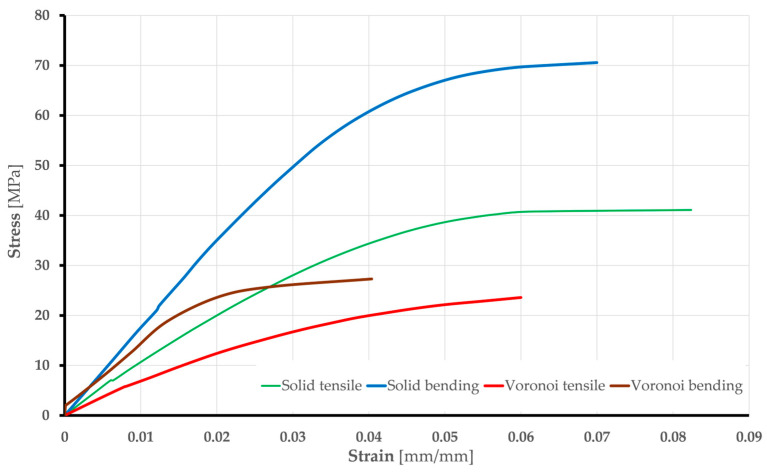
Stress vs. strain curves: numerical analysis for solid and Voronoi specimens.

Stress vs. strain curves obtained by numerical analysis for all specimens are shown in Figure 13.

#### Validation of Developed Numerical Models

**Figure 14:**
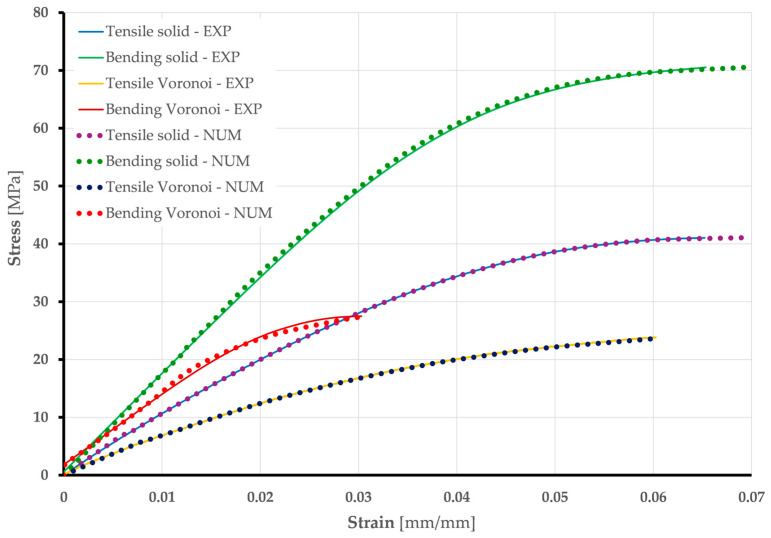
Stress vs. strain curves—comparison of experimental and numerical results for solid and Voronoi specimens.

The results presented above, which were obtained through numerical analyses, form the basis for the validation of the numerical models developed. The most important validation parameters are the comparison of stress vs. strain diagrams and deformation shapes from experimental and numerical analyses. Figure 14 shows that the experimental (true curve) and numerical stress vs. strain curves match well. Minor deviations in the bending of the Voronoi specimens are acceptable due to their stochastic geometry.

**Figure 15:**
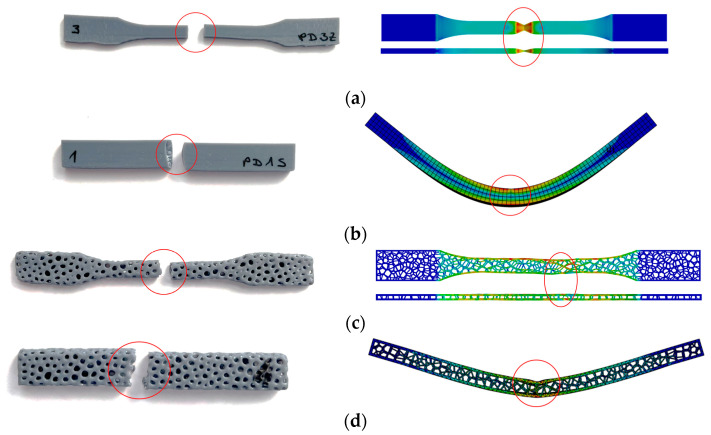
Deformed specimens—comparison of experimental (**left**) and numerical (**right**) results: (**a**) tensile—solid specimen, (**b**) bending—solid specimen, (**c**) tensile—Voronoi specimen, (**d**) bending—Voronoi specimen. Red circles represent fracture zone.

Figure 15 shows the appearance of the specimens after the experimental tests and the numerical analysis. The examination of the tensile specimens shows a remarkably good correlation of the necking positions. For the bending specimens, both the experimental and numerical analyses indicate that the maximum deformation occurs in the center of the specimens, which is in line with expectations.

## 5. Conclusions

Although thermoplastic PLA filaments have been well studied in the FDM field, the mechanical performance of PLA-based resins in DLP printing, especially for lattice structures, has not yet been sufficiently researched.

In this research, the tensile and bending behavior of solid and Voronoi specimens of PLA resin was analyzed, supported by validated numerical models, which agreed well with the experimental results. While Voronoi specimens exhibited lower tensile and flexural strength compared to solid specimens, they showed better stress distribution and material efficiency due to their porosity. Numerical analysis proved to be a cost-effective tool for predicting stress concentration, fracture initiation, and failure patterns, allowing early optimization of the design. The fracture analysis revealed brittle failure modes in both structures, with the Voronoi patterns showing characteristic fractures along the internal voids. Minor deviations between the designed and printed Voronoi models, due to swelling of the resin during polymerization, were observed but did not significantly affect the mechanical performance or structural integrity of the specimens.

Despite their lower strength, Voronoi lattices offer promising potential for biomedical immobilization devices and aerospace components where weight reduction and controlled porosity are critical. In future research, the plan is to systematically vary the printing parameters and geometry of Voronoi structure cells in order to evaluate their effect on the structural strength of the generated Voronoi structures.

## Figures and Tables

**Table 1:** DLP 3D printing process parameters.

Parameter	Value/Units
Layer height	0.05 mm
Bottom layer count	10
Exposure time	8 s
Bottom exposure time	50 s
Bottom lift distance	5 mm
Bottom lift speed	65 s

**Table 2:** Ratios for mass, force, and stroke.

Testing	*m_voro_* (g)	*m_solid_* (g)	*m_ratio_* (%)	*F_maxvoro_* (N)	*F_maxsolid_* (N)	*F_ratio_* (%)	*stroke_maxvoro_* (mm)	*stroke_maxsolid_* (mm)	*stroke_ratio_* (%)
Tensile	1.1	1.6	68.75	67.43	384.7	0.17	2.28	4.4	0.52
Bending	1.5	3.5	42.85	27.59	110.24	0.25	5.49	9.06	0.60

## Data Availability

Data can be requested via the corresponding author.

## References

[1] Dehghan S., Sattarpanah Karganroudi S., Echchakoui S., Barka N. (2025). The Integration of Additive Manufacturing into Industry 4.0 and Industry 5.0: A Bibliometric Analysis (Trends, Opportunities, and Challenges). Machines.

[2] Kumar S., Kumar R.A. (2025). Comprehensive Study on Additive Manufacturing Techniques, Machine Learning Integration, and Internet of Things-Driven Sustainability Opportunities. J. Mater. Eng. Perform..

[3] Ponis S., Aretoulaki E., Maroutas T.N., Plakas G., Dimogiorgi K. (2021). A Systematic Literature Review on Additive Manufacturing in the Context of Circular Economy. Sustainability.

[4] Nath S.D., Nilufar S. (2020). An Overview of Additive Manufacturing of Polymers and Associated Composites. Polymers.

[5] Saleh Alghamdi S., John S., Roy Choudhury N., Dutta N.K. (2021). Additive Manufacturing of Polymer Materials: Progress, Promise and Challenges. Polymers.

[6] Pagac M., Hajnys J., Ma Q.P., Jancar L., Jansa J., Stefek P., Mesicek J. (2021). A Review of vat photopolymerization technology: Materials, applications, challenges, and future trends of 3D printing. Polymers.

[7] Lublin D., Hao T., Malyala R., Kisailus D. (2024). Multiscale Mechanical Characterization of Biobased Photopolymers Towards Sustainable Vat Polymerization 3D Printing. RSC Adv..

[8] Pan Y., Zhou C., Chen Y. (2012). A Fast Mask Projection Stereolithography Process for Fabricating Digital Models in Minutes. ASME J. Manuf. Sci. Eng..

[9] Paral S.K., Jeng J.Y., Cheng Y.L., Lin D.Z. (2025). Novel vat design for liquid crystal display vat photopolymerization: Reduction of separation force and pixelated effect. Virtual Phys. Prototyp..

[10] Tumbleston J.R., Shirvanyants D., Ermoshkin N., Janusziewicz R., Johnson A.R., Kelly D., Chen K., Pinschmidt R., Rolland J.P., Ermoshkin A. (2015). Continuous liquid interface production of 3D objects. Science.

[11] Wada J., Wada K., Gibreel M., Wakabayashi N., Iwamoto T., Vallittu P.K., Lassila L. (2022). Effect of 3D Printer Type and Use of Protection Gas During Post-Curing on Some Physical Properties of Soft Occlusal Splint Material. Polymers.

[12] Chaudhary R., Fabbri P., Leoni E., Mazzanti F., Akbari R., Antonini C. (2023). Additive manufacturing by digital light processing: A review. Prog. Addit. Manuf..

[13] Shao S., Zhou Z. (2024). Design and Fabrication of lightweight structures using topology optimization techniques. Int. J. Mach. Tools Maint. Eng..

[14] Efstathiadis A., Symeonidou I., Tsongas K., Tzimtzimis E.K., Tzetzis D. (2023). Parametric Design and Mechanical Characterization of 3D-Printed PLA Composite Biomimetic Voronoi Lattices Inspired by the Stereom of Sea Urchins. J. Compos. Sci..

[15] Wang X., Xu X., Gu Y. (2022). Dynamic response of Voronoi structures with gradient perpendicular to the impact direction. Rev. Adv. Mater. Sci..

[16] Chen L., Pan Y., Chu X., Hui H., Wang X. (2023). Multiscale design and experimental verification of Voronoi graded stochastic lattice structures for the natural frequency maximization problem. Acta Mech. Sin..

[17] Liu R., Chen W., Zhao J. (2024). A Review on Factors Affecting the Mechanical Properties of Additively-Manufactured Lattice Structures. J. Mater. Eng. Perform..

[18] Taib N.-A.A.B., Rahman R., Huda D., Kuok K.K., Hamdan S., Bin Bakri M.K., Bin Julaihi M.R.M., Khan A. (2023). A review on poly lactic acid (PLA) as a biodegradable polymer. Polym. Bull..

[19] Wu Y., Gao X., Wu J., Zhou T., Nguyen T.T., Wang Y. (2023). Biodegradable Polylactic Acid and Its Composites: Characteristics, Processing, and Sustainable Applications in Sports. Polymers.

[20] Guessasma S., Stephant N., Durand S., Belhabib S. (2024). Digital Light Processing Route for 3D Printing of Acrylate-Modified PLA/Lignin Blends: Microstructure and Mechanical Performance. Polymers.

[21] Ligon S.C., Liska R., Stampfl J., Gurr M., Mülhaupt R. (2017). Polymers for 3D Printing and Customized Additive Manufacturing. Chem. Rev..

[22] Cahyono S.I., Sandi A., Salim U.A., Suyitno S., Arifvianto B., Saptoadi H., Mahardika M. (2024). Flame Retardant Additives in Polylactic Acid (PLA) Photopolymer Resin for 3D Printing Digital Light Processing (DLP). Appl. Mech. Mater..

[23] Golubović Z., Bojović B., Kirin S., Milovanović A., Petrov L., Anđelković B., Sofrenić I. (2024). Effect of Aging on Tensile and Chemical Properties of Polylactic Acid and Polylactic Acid-Like Polymer Materials for Additive Manufacturing. Polymers.

[24] Efstathiadis A., Symeonidou I., Tsongas K., Tzimtzimis E.K., Tzetzis D. (2023). 3D Printed Voronoi Structures Inspired by Paracentrotus lividus Shells. Designs.

[25] Hanon M.M., Dobos J., Zsidai L. (2021). The influence of 3D printing process parameters on the mechanical performance of PLA polymer and its correlation with hardness. Procedia Manuf..

[26] Hsueh M.-H., Lai C.-J., Chung C.-F., Wang S.-H., Huang W.-C., Pan C.-Y., Zeng Y.-S., Hsieh C.-H. (2021). Effect of Printing Parameters on the Tensile Properties of 3D-Printed Polylactic Acid (PLA) Based on Fused Deposition Modeling. Polymers.

[27] Ali Khan W., Hassan M., Ahmed I., Xiao M., Faraz M.I., Li K., Khan I., Muhammad R., Wu H., Hussain G. (2024). Insights into flexural and impact properties of polymer based materials printed through fused filament fabrication: Progress in the last decade. Int. J. Lightweight Mater. Manuf..

[28] Yalçın M.M. (2024). Flexural Behavior of 3D-Printed Carbon Fiber-Reinforced Nylon Lattice Beams. Polymers.

[29] Anand Kumar S., Shivraj Narayan Y., Chandrasekhar U., Yang L.J., Gowthaman S. (2019). Tensile Testing and Evaluation of 3D-Printed PLA Specimens as per ASTM D638 Type IV Standard. Innovative Design, Analysis and Development Practices in Aerospace and Automotive Engineering (I-DAD 2018). Lecture Notes in Mechanical Engineering.

[30] Tavuz K.A., Al-Haj Husain N., Mätzener K.J., Ateş M.M., Eyüboğlu T.F., Özcan M. (2025). Evaluation of flexural strength of additively manufactured resin materials compared to auto-polymerized provisional resin with and without hydrothermal aging. J. Mech. Behav. Biomed. Mater..

[31] Li X., McMains S. (2023). A Voronoi diagram approach for detecting defects in 3D printed fiber-reinforced polymers from microscope images. Comp. Vis. Media.

[32] (2024). SolidWorks Premium 2024 SP05.

[33] (2012). Plastics—Determination of Tensile Properties—Part 2: Test Conditions for Moulding and Extrusion Plastics.

[34] (2019). Plastics—Determination of Flexural Properties.

[35] Hayes B.S., Gammon L.M. (2010). Optical Microscopy of Fiber-Reinforced Composites 2010.

[36] Ansys^®^ Academic Research Mechanical, Release 2025R01, License No. 670265.

[37] Bassani R., Levita G., Meozzi M., Palla G. (2001). Friction and wear of epoxy resin on inox steel: Remarks on the influence of velocity, load and induced thermal state. Wear.

[38] Palaniandy L., Ismail K.I., Yap T.C. (2023). Trbological Behaviour of 3D printed Polylactic Acid (PLA) Sliding Against Steel at Different Sliding Speed. J. Phys. Conf. Ser..

[39] Fouly A., Assaifan A.K., Alnaser I.A., Hussein O.A., Abdo H.S. (2022). Evaluating the Mechanical and Tribological Properties of 3D Printed Polylactic-Acid (PLA) Green-Composite for Artificial Implant: Hip Joint Case Study. Polymers.

[40] Golubović Z., Danilov I., Bojović B., Petrov L., Sedmak A., Mišković Ž., Mitrović N. (2023). A Comprehensive Mechanical Examination of ABS and ABS-like Polymers Additively Manufactured by Material Extrusion and Vat Photopolymerization Processes. Polymers.

